# The *SAP* function in pistil development was proved by two allelic mutations in Chinese cabbage (*Brassica rapa* L. ssp. *pekinensis*)

**DOI:** 10.1186/s12870-020-02741-5

**Published:** 2020-11-30

**Authors:** Shengnan Huang, Wenjie Liu, Junjie Xu, Zhiyong Liu, Chengyu Li, Hui Feng

**Affiliations:** Department of Horticulture, Shenyang Agricultural University, 120 Dongling Road, Shenhe District, Shenyang, 110866 China

**Keywords:** Chinese cabbage, Female sterility, *STERILE APETALA*, RNA-Seq, EMS mutagenesis

## Abstract

**Background:**

Pistil development is a complicated process in plants, and female sterile mutants are ideal material for screening and cloning pistil development-related genes. Using the female sterile mutant (*fsm1*), *BraA04g009730.3C* was previously predicted as a candidate mutant gene encoding the STERILE APETALA (SAP) transcriptional regulator. In the current study, a parallel female sterile mutant (*fsm2*) was derived from EMS mutagenesis of a Chinese cabbage DH line ‘FT’ seeds.

**Results:**

Both *fsm2* and *fsm1* mutant phenotypes exhibited pistil abortion and smaller floral organs. Genetic analysis indicated that the phenotype of mutant *fsm2* was also controlled by a single recessive nuclear gene. Allelism testing showed that the mutated *fsm1* and *fsm2* genes were allelic. A single-nucleotide mutation (G-to-A) in the first exon of *BraA04g009730.3C* caused a missense mutation from GAA (glutamic acid) to GGA (glycine) in mutant *fsm2* plants. Both allelic mutations of *BraA04g009730.3C* in *fsm1* and *fsm2* conferred the similar pistil abortion phenotype, which verified the *SAP* function in pistil development. To probe the mechanism of *SAP*-induced pistil abortion, we compared the mutant *fsm1* and wild-type ‘FT’ pistil transcriptomes. Among the 3855 differentially expressed genes obtained, 29 were related to ovule development and 16 were related to organ size.

**Conclusion:**

Our study clarified the function of *BraA04g009730.3C* and revealed that it was responsible for ovule development and organ size. These results lay a foundation to elucidate the molecular mechanism of pistil development in Chinese cabbage.

**Supplementary Information:**

The online version contains supplementary material available at 10.1186/s12870-020-02741-5.

## Background

Female sterility refers to the phenomenon in which pistil fertility is reduced or completely aborted due to the abnormal development of female organs in plants. The pistil structure is complex, and an abnormal female organ development may lead to female sterility in the sporophyte and gametophyte stages. According to the specific period of pistil abortion, female plant sterility can be divided into three types: (1) abnormal pistil, (2) abnormal ovule, and (3) abnormal egg cell [1]. Sterile female plants are highly useful in studying the developmental mechanism and genetic breeding of female organs in higher plants [2–4].

Among the floral organs, the pistil has the most complex structure, and its reproductive growth and development processes are regulated by a large number of transcription factors and functional genes [5–7]. Several genes regulating pistil development and physiological and biochemical changes during pistil abortion have been identified through mapping and cloning of female sterile mutant genes in *Arabidopsis thaliana*, rice, cotton, maize, and rapeseed, leading to a gradual understanding of the morphological model and genetic regulation of pistil development [8–12]. High-throughput transcriptome sequencing of female sterile mutants enables to explore the metabolic pathway changes that occur during pistil abortion at the transcriptional level; screening of key genes regulating pistil development is helpful in studying pistil and ovule development, as well as the genetic regulation mechanism [13–17].

Pistil development is a complex process controlled by multiple genes. Coen and Meyerowitz [18] proposed the ABC model of floral organ development, then Colombo et al. [19] and Theissen [20] extended the ABC model to the ABCD and ABCDE models. Among these, class D genes regulate ovule development, and most genes in the ABCDE model belong to the MADS-box gene family [21]. There are several collateral homologous genes in the MADS-box gene family that jointly regulate flower development [19, 22–24]. In *A. thaliana*, the ovule identity genes identified include *AGAMOUS* (*AG*), *BELL1* (*BEL1*), *SEEDSTICK* (*STK*), SHATTERPROOF1*/2* (*SHP1/SHP2*), *CUP-SHAPED COTYLEDON3* (*CUC3*), and *PRETTY FEW SEEDS2* (*PFS2*); these genes are closely related to ovule morphogenesis and encode proteins that contain the conserved MADS-box functional domains [25–30]. Genes associated with ovule primordia formation include *AINTEGUMENTA* (*ANT*), *WUSCHEL* (*WUS*), *NOZZLE* (*NZZ*), *INNER NO OUTER* (*INO*), *BEL1,* and *PHABULOSA* (*PHB*) [4, 31–34]. In addition, several genes related to integument development have been isolated and divided into two categories: one category controls the early development of integument and includes *HUELLENLOS* (*HLL*), *ANT*, *NZZ*, and *INO* [35–38]; the other controls the later development of integument and includes *SHORT INTEGUMENT1* (*SIN1*), *SUPERMAN* (*SUP*), *STRUBBELIG* (*SUB*), *ARABIDOPSIS CRINKLY4* (*ACR4*), *ABERRANT TESTA SHAPE* (*ATS*), *KANADI1/2* (*KAN1/2*), *UNICORN* (*UCN*), and *TSO1* [39–43].

In our previous study, we obtained a female sterile mutant *fsm* (namely *fsm1* here) by isolated microspore culture combined with ethyl methanesulfonate (EMS) mutagenesis of the Chinese cabbage (*Brassica rapa* L. ssp. *pekinensis*) double haploid (DH) line ‘FT’ [44]. Pistil abortion in the *fsm1* mutant was caused by abnormal ovules and *BraA04g009730.3C* (version 3.0) was presumed to be the candidate gene in the *fsm1* mutant based on map-based cloning and whole-genome re-sequencing [12]. *BraA04g009730.3C* encodes STERILE APETALA (SAP), a transcriptional regulator that plays an important role in floral organ development. In *A. thaliana*, *SAP* not only regulates flower and ovule development [45], but also controls organ size by affecting cell proliferation [46].

In this study, the DH line ‘FT’ was used as mutagenic material; germinating ‘FT’ seeds were treated with EMS solution to develop another parallel female sterile mutant (*fsm2*) whose phenotype was consistent with that of mutant *fsm1*. Allelism testing indicated that the mutant genes *fsm1* and *fsm2* were allelic. These two parallel mutants were used to verify the function of *BraA04g009730.3C*. To investigate the potential mechanism of ovule development, RNA-sequencing was used to compare the pistil transcriptome of mutant *fsm1* and wild-type ‘FT’ plants. Genes related to ovule development and organ size were identified and screened, laying a foundation to further reveal the pistil abortion mechanism in Chinese cabbage.

## Results

### Comparison of morphological characteristics between the *fsm1* and *fsm2* mutants

**Fig. 1:**
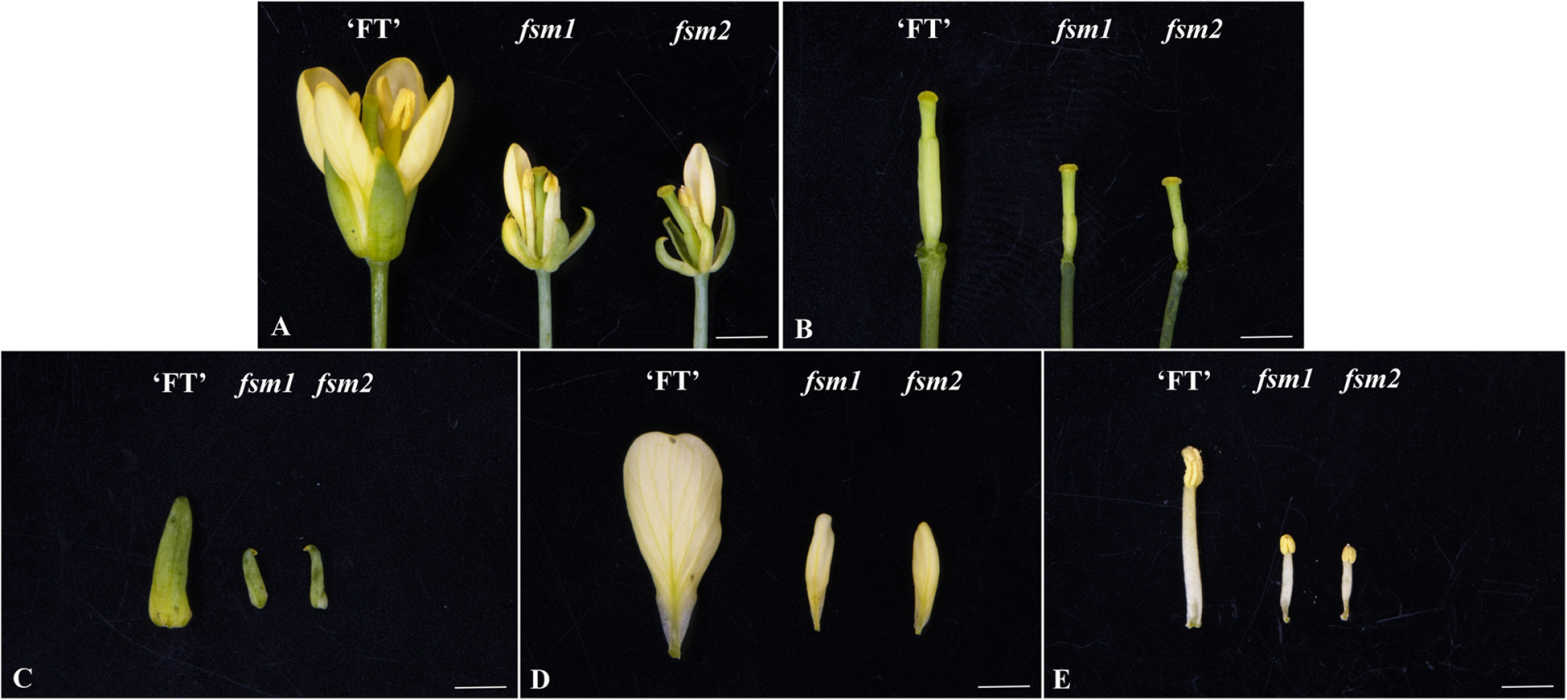
Morphological characterization of the wild-type ‘FT’ plants and female-sterile *fsm1* and *fsm2* mutants*.* **a**. Flowers of the wild-type ‘FT’ plants and *fsm1* and *fsm2* mutants. **b**. Pistils of the wild-type ‘FT’ plants and *fsm1* and *fsm2* mutants. **c**. Sepals of the wild-type ‘FT’ plants and *fsm1* and *fsm2* mutants. **d**. Petals of the wild-type ‘FT’ plants and *fsm1* and *fsm2* mutants. **e**. Stamens of the wild-type ‘FT’ plants and *fsm1* and *fsm2* mutants. Scale bar: 1 mm

The *fsm2* mutant phenotype was highly consistent with that of the *fsm1* mutant. Compared with the wild-type ‘FT’ plants, the mutant *fsm2* plants exhibited pistil abortion. As shown in Fig. 1b, the ovary was thin and short. In addition, the four-whorled floral organs of the mutant *fsm2* plants were significantly smaller those of the wild-type plants (Fig. 1).

As shown in Table 1, when a mutant *fsm2* plant was used as the female parent, irrespective of whether its pollen or foreign (wild-type ‘FT’) pollen was employed as the male parent, no seed was harvested from the offspring. However, when a mutant *fsm2* plant was used as the male parent, seeds could be collected from the offspring. Therefore, stamen fertility was normal but the pistil was abortive. Furthermore, female sterility of the *fsm2* mutant was stable.

**Table 1:** Seed-setting rates of mutant *fsm2*

Generation	No. of pollinated flower buds	No. of harvested seeds	No. of seeds per bud
*fsm2* × *fsm2*	60	0	0
*fsm2* × ‘FT’	60	0	0
‘FT’ × *fsm2*	60	657	10.95

### Genetic analysis of mutant *fsm2*

When a wild-type ‘FT’ plant was used as the female parent and the *fsm2* mutant was used as the male parent for hybridization, the phenotype of all plants was consistent with that of the wild-type ‘FT’ in the F_1_ generation. In the F_2_ generation, the segregation ratio was 3:1, whereas the segregation ratio was approximately 1:1 in the BC1 generation (F_1_ × *fsm2*). These results suggest that the *fsm2* mutant phenotype was controlled by a single recessive nuclear gene (Table 2).

**Table 2:** Genetic analysis of the *fsm2* mutant in Chinese cabbage

Generation	‘FT’	*fsm2*	Total	Segregation Ratio	Expected Ratio	Chi-square (χ2)
P_1_ (‘FT’)	30	0	30			
P_2_ (*fsm2*)	0	30	30			
F_1_ (P_1_ × P_2_)	200	0	200			
F′_1_ (P_2_ × P_1_)	0	0	0			
BC_1_ (F_1_ × ‘FT’)	193	0	193			
BC_1_ (F_1_ × *fsm2*)	98	88	186	1.11: 1	1:1	2.15
F_2_	172	53	225	3.25: 1	3:1	0.81

### Allelism testing

The reciprocal cross of both F_1_ (‘FT’ × *fsm1*) and F_1_ (‘FT’ × *fsm2*) exhibited character segregation. In the offspring, the segregation ratios of the wild-type-to-mutant plants were 137:32 (χ^2^ = 1.24 < χ^2^_0.05, 1_ = 3.84) and 147:45 (χ^2^ = 0.09 < χ^2^_0.05, 1_ = 3.84), respectively, consistent with the 3:1 segregation ratio. These results indicated that the mutant genes *fsm1* and *fsm2* were allelic and caused by mutations in the same gene.

### *BraA04g009730.3C* clone in mutant *fsm2*

**Fig. 2:**
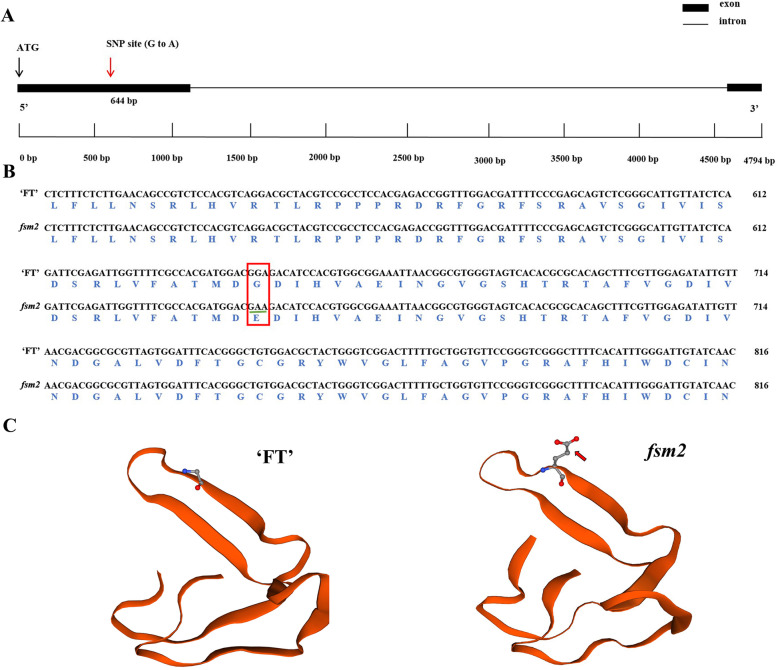
Comparison of *BraA04g009730.3C* gene structure and sequence alignment between wild-type ‘FT’ and mutant *fsm2* plants. **a**. Gene structure of *BraA04g009730.3C* and the single nucleotide polymorphism (SNP) site. **b**. Alignment of *BraA04g009730.3C* encoding sequences and amino acid sequences. The green underline and red frame show the sites where the SNP and the non-synonymous mutation occurred, respectively. **c**. Partial three-dimensional structure of *BraA04g009730.3C* in wild-type (left) and mutant *fsm2* (right). Amino acid sequences include the SNPs. The red arrow represents different amino acid conformations at the mutation site

A *BraA04g009730.3C* clone in mutant *fsm2* plants showed a single nucleotide mutation (G-to-A, A04: 7543809) in the first exon, causing an amino acid change from glutamic acid (G) to glycine (E), which differed from the mutation site of the *fsm1* mutant (Fig. 2a, b). The three-dimensional structures of the proteins showed that the amino acid conformation between the wild-type ‘FT’ and mutant *fsm2* were different at the mutant site (Fig. 2c). These results indicated that the two allelic mutations in *BraA04g009730.3C* conferred the similar pistil abortion phenotype and verified *SAP* function in pistil development.

### Illumina paired-end sequencing and global data analysis

The *fsm1* and *fsm2* plants are allelic mutants with different mutations in *BraA04g009730.3C* that encode a SAP transcriptional regulator. To examine the possible pathways through which *SAP* may regulate pistil development in the *fsm* plants, a comparative transcriptome analysis of ‘FT’ and *fsm1* pistils was conducted. A total of 128,643,734 and 130,558,248 clean reads were obtained from the three biological replicates of the ‘FT’ and *fsm1* plants, respectively. Of the total clean reads, the percentage of reads mapped to the reference genome ranged from 90.66 to 91.76% in the six libraries and 97.48 to 97.88% of the mapped reads were matched to unique genomic locations (Table 3) that could be used for the differential analysis of gene expression between the ‘FT’ and *fsm1* plants*.*

**Table 3:** Read statistics based on the RNA-Seq data of six libraries of the ‘FT’ and mutant *fsm1* plants

Summary	FT1	FT2	FT3	M1	M2	M3
Total clean reads	42,746,476	41,561,500	44,335,758	42,295,428	45,020,978	43,241,842
Total base pairs	6,411,971,400	6,234,225,000	6,650,363,700	6,344,314,200	6,753,146,700	6,486,276,300
Total mapped reads	39,033,708 (91.31%)	38,088,951 (91.64%)	40,682,504 (91.76%)	38,770,778 (91.67%)	40,816,520 (90.66%)	39,425,925 (91.18%)
Uniquely mapped reads	38,117,525 (97.65%)	37,249,014 (97.79%)	39,755,549 (97.72%)	37,950,226 (97.88%)	39,788,236 (97.48%)	38,451,819 (97.53%)
Multiple mapped reads	916,183 (2.35%)	839,937 (2.21%)	926,955 (2.28%)	820,552 (2.12%)	1,028,284 (2.52%)	974,106 (2.47%)

Note: Total mapped reads are the sum of uniquely matched reads and multiple mapped reads

In addition, 24,494 (FT1), 24,668 (FT2), 24,863 (FT3), 24,918 (M1), 24,860 (M2), and 25,418 (M3) genes expressed were generated from these six libraries, respectively (Additional file 1: Table S1).

### Differentially expressed genes (DEGs) between the ‘FT’ and *fsm1* plants

A comparison of ‘FT’ vs. *fsm1* plants revealed 3855 DEGs, of which 2356 were upregulated and 1499 were downregulated (Additional file 2: Table S2). The number of upregulated DEGs was significantly higher than that of the downregulated DEGs in the *fsm1* mutant. Of the DEGs, 106 were specifically expressed, with 21 and 85 specifically expressed in the ‘FT’ and *fsm1* plants, respectively (Additional file 3: Table S3).

### Functional enrichment analysis of DEGs

The GO functional enrichment analysis was performed to identify the biological functions of DEGs. We identified 1304 enriched GO terms. Of these, 768, 126, and 410 GO terms were in the “biological process,” “cellular component,” and “molecular function” categories, respectively (Additional file 4: Fig. S1). Two GO terms related to flower development were identified (“flower development” (GO: 0009908; four DEGs) and “regulation of flower development” (GO: 0009909; one DEG)). In addition, numerous GO terms associated with plant hormone metabolism were also identified, including “response to hormone” (GO: 0009725; 21 DEGs), “hormone-mediated signaling pathway” (GO: 0009755; one DEG), and “response to auxin” (GO: 0009733; 16 DEGs). The significantly enriched GO terms are shown in Additional file 5: Table S4.

**Fig. 3:**
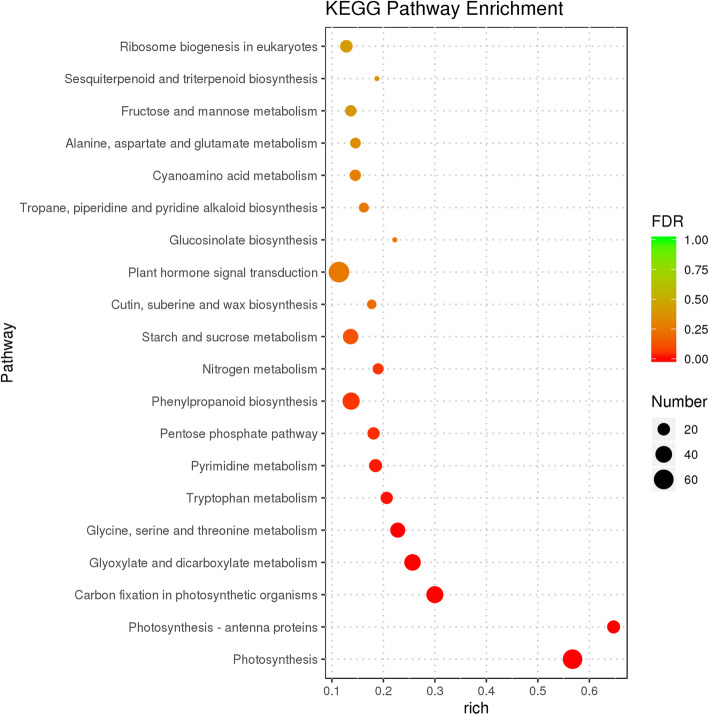
Twenty most significantly enriched KEGG metabolic pathways

**Fig. 4:**
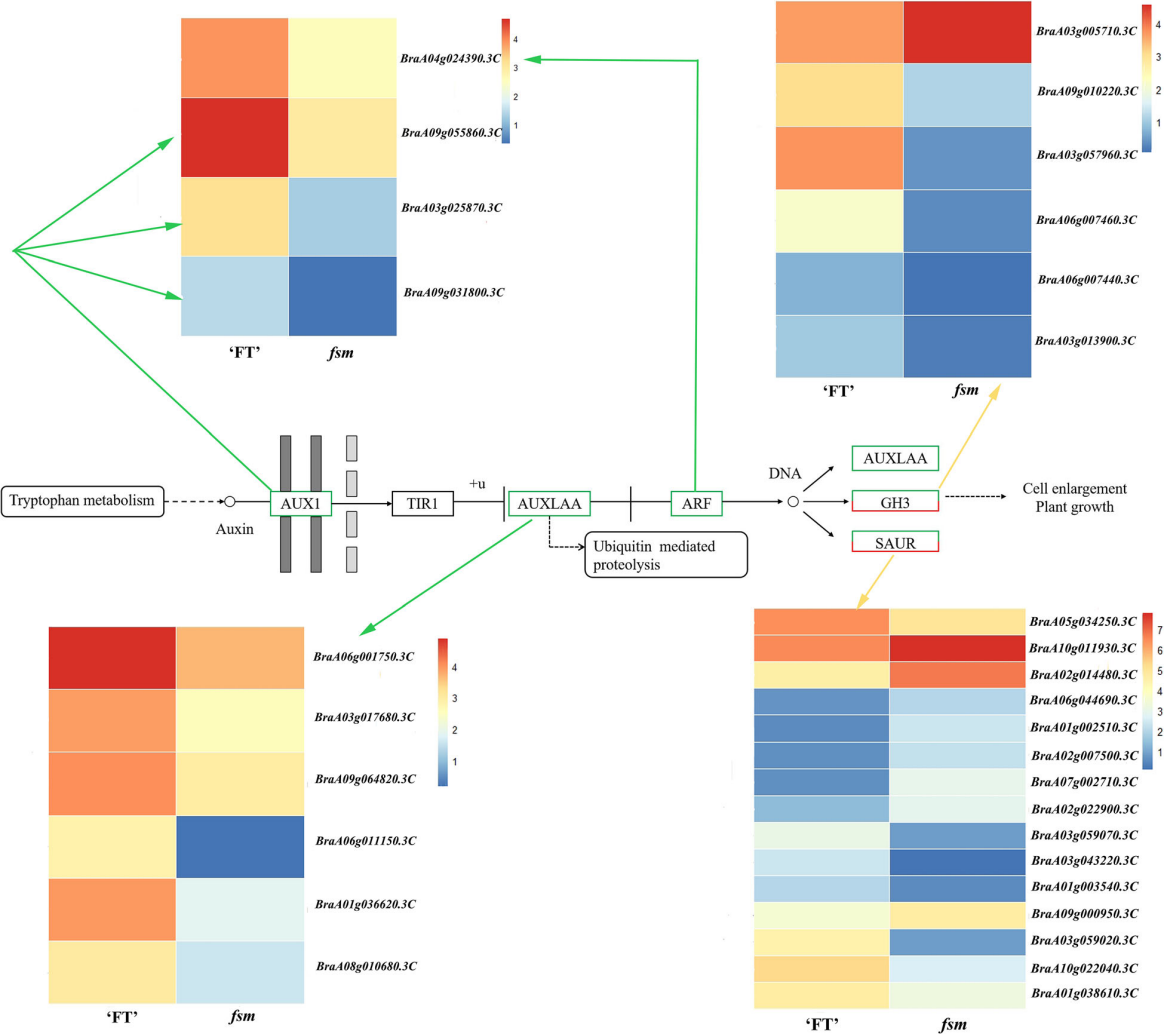
Expression of differentially expressed genes involved in the auxin signal transduction pathway. Note: The bar on the right represents the relative expression values. Gene expression is measured by counting the log_2_-based fragments per kilobase of transcript per million read values. Green arrows indicate genes that were downregulated and yellow arrows indicate genes that were both upregulated and downregulated

The KEGG pathway analysis was performed to confirm the genes involved in metabolic or signal transduction pathways. We identified 117 enriched KEGG pathways. The 20 most significantly enriched KEGG pathways are shown in Fig. 3 and Additional file 6: Table S5. Of these, the “plant hormone signal transduction” pathway (KO04075) was significantly enriched, with 67 DEGs grouped into the auxin (IAA), cytokinin (CK), gibberellin (GA), abscisic acid (ABA), ethylene (ETH), brassinosteroid (BR), jasmonic acid (JA), and salicylic acid (SA) signal transduction pathways. Previous studies have shown that the plant hormones IAA, CK, ETH, GA, JA, and BR can influence the pistil development [11, 47, 48]. Of these, 31 DEGs were enriched in the IAA signal transduction pathway, followed by BR (7 DEGs), JA (5 DEGs), ETH (2 DEGs), CK (1 DEG), and GA (1 DEG) (Additional file 7: Table S6). The DEGs involved in the IAA signal transduction process included auxin1 (*AUX1*), auxin/indole-3-acetic acid (*AUX/IAA*), auxin response factors (*ARF*), *GH3,* and small auxin up RNA (*SAUR*); the majority of these genes were downregulated in the *fsm1* mutant (Fig. 4).

### Analysis of DEGs related to ovule development and organ size

Among the DEGs, 29 were related to ovule development. Of these, the *AGAMOUS-like* (*AGL*) genes (*BraA09g048000.3C*, *BraA03g051930.3C*, *BraA03g058700.3C*, *BraA09g006770.3C*, and *BraA06g039170.3C*) were involved in ovule morphogenesis; genes encoding *ANT* (*BraA03g061040.3C* and *BraA05g011060.3C*), *CUC3* (*BraA07g039980.3C*), and *WUS* (*BraA03g038230.3C*, *BraA05g011060.3C*, *BraA05g029540.3C*, and *BraA10g022260.3C*) were involved in ovule primordia formation; and genes encoding *ANT*, *BEL1* (*BraA03g000480.3C*, *BraA04g013970.3C*, *BraA03g058930.3C*, *BraA04g025560.3C*, *BraA03g018820.3C*, *BraA10g033530.3C*, *BraA07g027760.3C*, *BraA05g009430.3C*, *BraA04g017450.3C*, and *BraA07g038990.3C*), *TSO1* (*BraA09g005800.3C*, *BraA01g021830.3C*, and *BraA05g024430.3C*), *SUB* (*BraA06g007870.3C* and *BraA06g001180.3C*), *SUP* (*BraA05g002900.3C*), and *KAN1* (*BraA02g031490.3C*) were involved in integument development. These genes may be associated with pistil abortion in the *fsm1* mutant.

Compared with the wild-type ‘FT’ plants, the *fsm1* mutant showed pistil abortion, and the four-whorled floral organs were significantly small. We identified a number of genes related to organ size regulation including the following: *ANT*, *AUXIN-REGULATED GENE INVOLVED IN ORGAN SIZE* (*ARGOS*; *BraA09g049790.3C*), *TEOSINTE BRANCHED1/CYCLOIDEA/PCF* (*TCP*; *BraA02g042770.3C*, *BraA05g005380.3C*, *BraA05g032060.3C*, *BraA01g036950.3C*, *BraA07g034590.3C*, *BraA08g023460.3C*, *BraA07g030260.3C*, *BraA03g036760.3C*, and *BraA05g041050.3C*) and *AINTEGUMENTA-LIKE* (*AIL*; *BraA06g029000.3C*, *BraA03g004320.3C*, *BraA10g027380.3C*, and *BraA02g012300.3C*). Most of these genes were downregulated in the *fsm1* mutant. This downregulation may play a role in regulating floral organ size in the *fsm1* mutants.

### Analysis of gene expression patterns by quantitative reverse-transcription PCR (RT-qPCR)

**Fig. 5:**
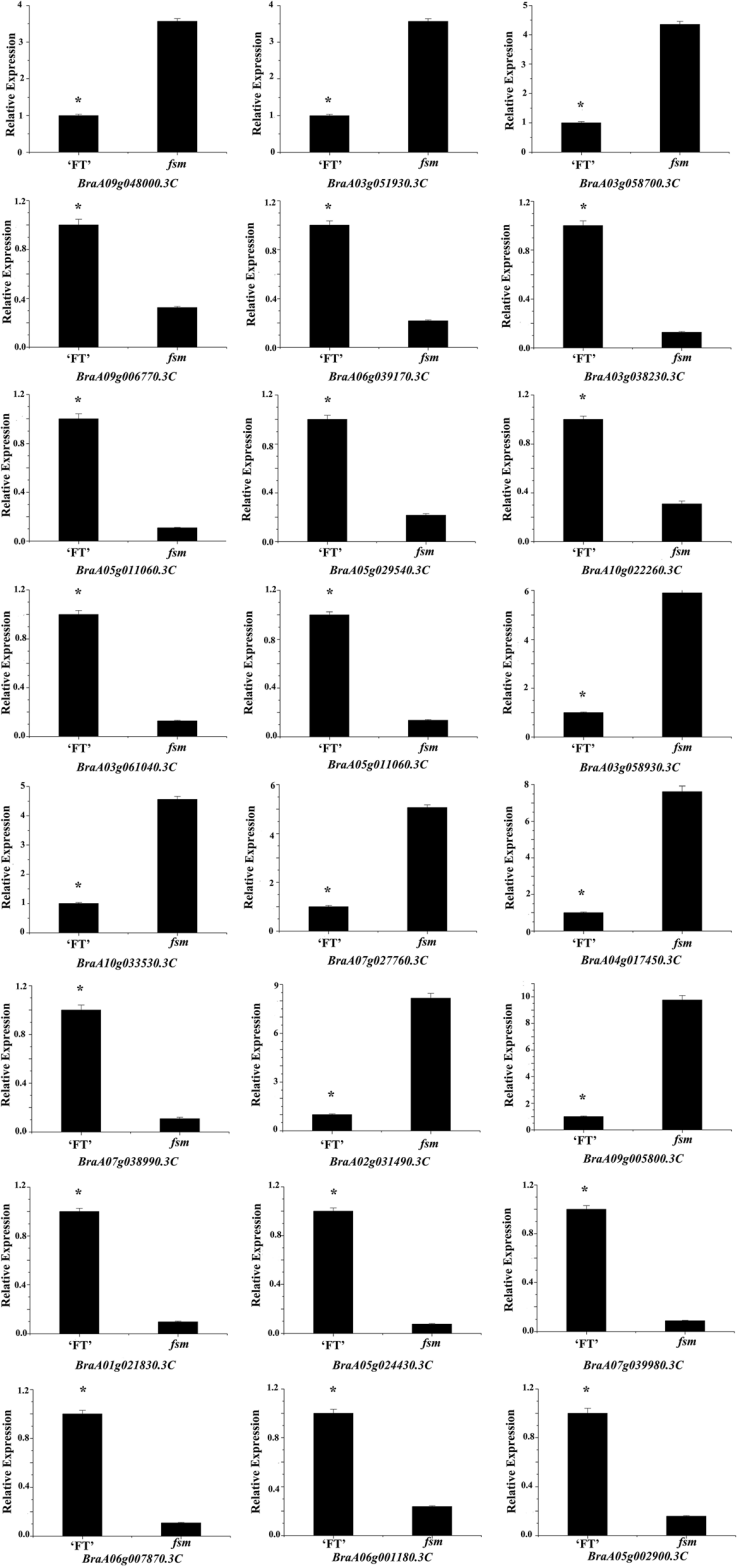
RT-qPCR analysis of gene expression patterns. * indicates a significant difference at the 0.05 level determined by *t*-test

To further confirm DEG expression patterns, 24 DEGs related to ovule development were selected for the RT-qPCR analysis including genes encoding *AGL* (*BraA09g048000.3C*, *BraA03g051930.3C*, *BraA03g058700.3C*, *BraA09g006770.3C*, and *BraA06g039170.3C*), *WUS* (*BraA03g038230.3C*, *BraA05g011060.3C*, *BraA05g029540.3C*, and *BraA10g022260.3C*), *ANT* (*BraA03g061040.3C* and *BraA05g011060.3C*), *BEL1* (*BraA03g058930.3C*, *BraA10g033530.3C*, *BraA07g027760.3C*, *BraA04g017450.3C*, and *BraA07g038990.3C*), *KAN1* (*BraA02g031490.3C*), *TSO1* (*BraA09g005800.3C*, *BraA01g021830.3C*, and *BraA05g024430.3C*), *CUC3* (*BraA07g039980.3C*), *SUB* (*BraA06g007870.3C* and *BraA06g001180.3C*), and *SUP* (*BraA05g002900.3C*). As shown in Fig. 5, the gene expression patterns showed a tendency similar to those detected by RNA-Seq, indicating the reliability of our transcriptome analysis.

## Discussion

In our previous study, we developed a female sterile mutant (*fsm1*) using isolated microspore culture combined with EMS mutagenesis in DH line ‘FT’ Chinese cabbage. *BraA04g009730.3C* was predicted as the candidate gene in mutant *fsm1* that encodes a SAP transcriptional regulator; the coding sequence of *BraA04g009730.3C* is 1374 bp long [12]. In the present study, ‘FT’ seeds were also employed as the mutagenic material and germinating ‘FT’ seeds were treated with EMS solution to develop another parallel female sterile mutant (*fsm2*) that displayed a phenotype consistent with the *fsm1* mutant. Allelism testing and the gene cloning analysis indicated that *fsm1* and *fsm2* were allelic and caused by mutations in the same gene, which verified the *SAP* function in pistil development. To probe the pistil abortion mechanism caused by *SAP*, comparative transcriptome sequencing of the pistil of the *fsm1* mutant and wild-type ‘FT’ plants was conducted and the genes related to ovule development and organ size were identified. These results lay a foundation to reveal the mechanism of pistil abortion caused by the *SAP* gene in Chinese cabbage.

In plants, gene overexpression, RNA interference, gene knockout, site-directed mutagenesis, gene trapping, and biochip technology are commonly used methods for studying gene function. However, all these methods depend on the complete transgenic technology system, and they are time consuming. In contrast, allelism detection of mutant genes in different mutants with similar phenotype is a believable approach to verify whether they are controlled by the allelic genes, and can be applied to clarify gene function. Zhang et al. [49] identified two round-leaf mutants, *rl*-*1* and *rl*-*2*, with a similar smooth leaf margin from a cucumber EMS mutagenic population. The map-based cloning strategy combined with a modified MutMap method suggested that *CsPID* (encoding a serine/threonine protein kinase) was the most likely candidate for *rl*-*1*. An allelism test of a cross between the *rl*-*1* F_1_ (*rl*-*1* × wild-type CCMC) and *rl*-*2* F_1_ (*rl*-2 × wild-type CCMC) plants showed that the segregation ratio of normal leaf shape-to-round leaf shape was approximately 3:1, suggesting that *rl*-*1* and *rl*-*2* were allelic mutants with mutations in the same gene. These results suggest that *CsPID* is the gene responsible for the round leaf phenotype. Similar results have been reported in maize [11] and rice [50]. In the present study, the separation ratio of reciprocal crosses between the F_1_ (‘FT’ × *fsm1*) and F_1_ (‘FT’ × *fsm2*) plants was 3:1, suggesting that the *fsm1* and *fsm2* mutants had allelic mutations in the same gene. Based on our previous study [12], an SNP (C-to-A) occurred in the first exon (A04: 7544007) of *BraA04g009730.3C*, resulting in a premature stop codon in mutant *fsm1*; another SNP (G-to-A) was located in the first exon (A04: 7543809) of *BraA04g009730.3C*, causing a non-synonymous mutation in mutant *fsm2*. These results indicated that the allelic mutations in *BraA04g009730.3C* encoding an SAP transcriptional factor were responsible for pistil development in *fsm1* and *fsm2* mutants.

The SAP transcription factor was initially identified in *A. thaliana*, where *SAP* is essential for flower development. The *sap* mutant exhibited serious abnormalities in inflorescence, flower, and ovule development [45]. SAP encodes an F-box protein, which is a component of the SKP1/Cullin/ F-box (SCF) E3 ubiquitin ligase complex [46]. *SAP* affects organ size by regulating cell proliferation in *A. thaliana* [51]. In the genus *Capsella*, decreased SAP activity can result in small petals by shortening the cell proliferation period and reducing the number of petal cells [52]. Yang et al. [53] found a cucumber littleleaf (*ll*) mutant that exhibited smaller organ sizes and more lateral branches. Identification of the major-effect quantitative trait loci showed that *LL* in cucumber is an ortholog of *Arabidopsis SAP* and that they play similar roles in organ size control. In the present study, the *fsm1* and *fsm2* mutants presented an identical phenotype, exhibiting pistil abortion and small floral organs compared with the wild-type ‘FT’ plants, demonstrating that the *SAP* gene was involved in the mutant phenotype of Chinese cabbage.

**Fig. 6:**
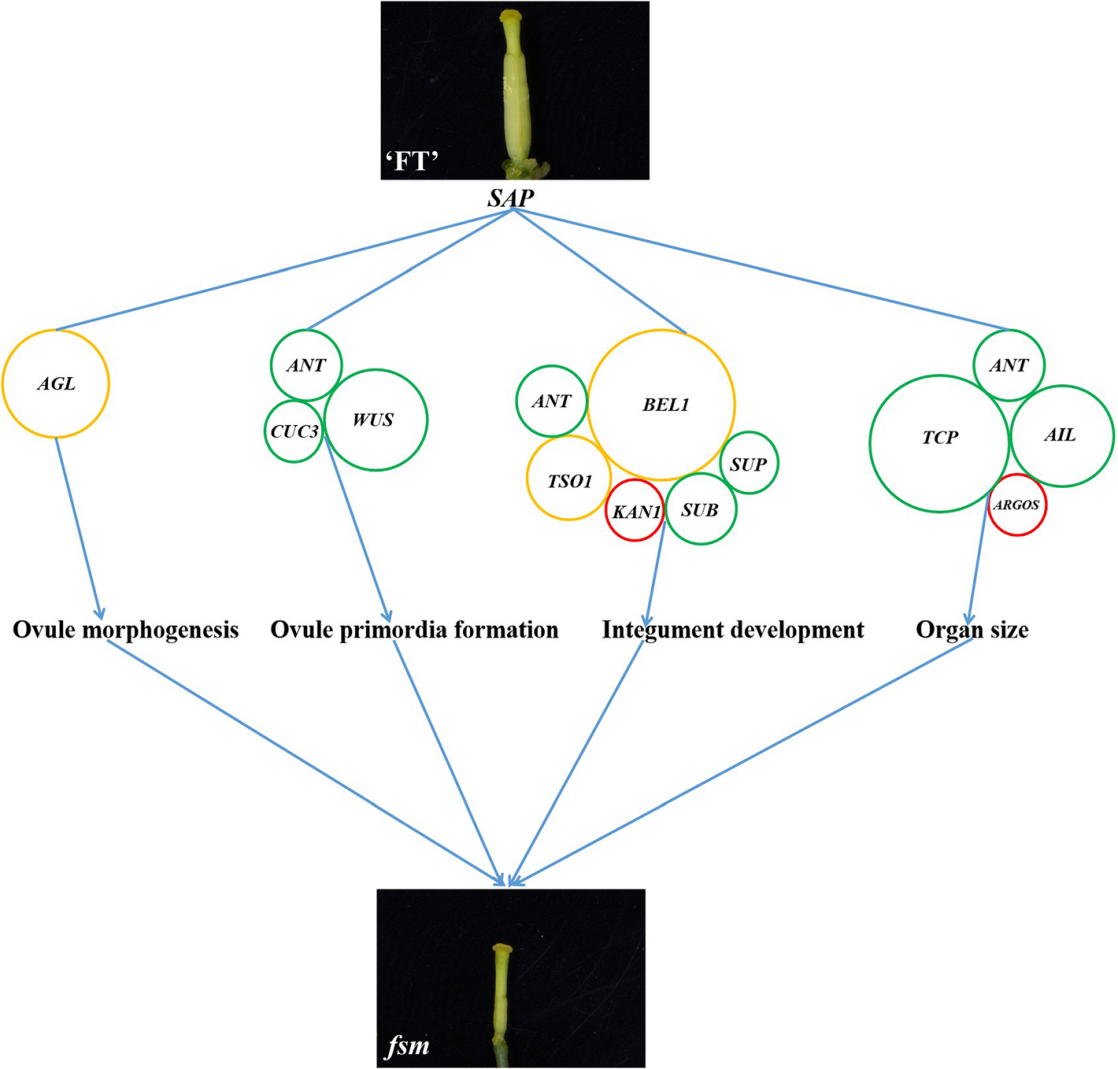
Possible *SAP*-mediated regulatory pathways involved in *fsm* mutants pistil development. The size of the circle represents the number of genes. Red and green indicate upregulated and downregulated genes, respectively. Yellow indicates genes that were both upregulated and downregulated

Our previous study showed that an SNP was identified in *BraA04g009730.3C*, resulting in a premature stop codon in mutant *fsm1*, and *BraA04g009730.3C* expression had no obvious difference between the ‘FT’ and *fsm1* mutant plants [12]. In this study, another SNP was located in *BraA04g009730.3C*, causing a non-synonymous mutation in mutant *fsm2*. We further analyzed the protein three-dimensional structures, and the results showed that the amino acid conformation were different at the mutant site between the wild-type ‘FT’ and mutant *fsm2*. The abnormal function of *SAP* in the *fsm* mutants was mainly manifested in amino acid coding. Given this, we speculated that the single nucleotide variation had no influence on *BraA04g009730.3C* expression, however, the variant protein may affect the expression of its downstream genes, eventually leading to phenotypic variation. We also demonstrated that mutant *fsm1* pistil abortion was caused by abnormal ovule development [12, 44]. To further investigate the potential mechanism of ovule development induced by the *SAP* gene, we used RNA-Seq to compare the pistil transcriptome of the mutant *fsm1* and wild-type ‘FT’ plants. There are two main regulatory pathways of pistil development in plants. In the first pathway, *WUS*-*AG*-related genes jointly regulate pistil development and in the second pathway, the *KNOXI* genes regulate pistil development. Of these, the first pathway is the main regulatory pathway [54, 55], suggesting that the *AG* genes play essential roles in the pistil development process. Studies have shown that SAP negatively regulates *AG* expression and that they jointly determine floral organ differentiation [45]. *AG* belongs to the MADS-box gene family and plays an important role in ovule development; its activity also contributes to ovule morphogenesis [21, 29, 56]. As important regulatory genes in ovule development, *AG* homologs also play essential roles in pistil formation [57]. For example, *AGL11* plays an important regulatory role in ovule development [58], *AGL8* and *AGL19* play vital roles in regulating floral transition and female sterility [16, 59], and *AGL62* can stimulate nucellus degeneration [60]. The homeobox gene *WUS* plays an essential role in regulating ovule development; it is mainly expressed in the nucellus of ovule primordia and is required for integument initiation [33]. *ANT* is a member of the AP2/ERF transcription factor family, which is involved in ovule development and ovule primordia formation [61, 62]. *ANT* mutation results in failed ovule integument formation, which then results in abnormal ovule development and female sterility [63, 64]. *BEL1* is a homeodomain transcription factor that can control ovule patterning, particularly in determining integument identity and development; ovules develop a single integument-like structure in *bel1* mutants [25, 65]. *CUC3* is a putative NAC-domain transcription factor member of the *CUC* gene family [27]. *CUC1*, *CUC2,* and *CUC3* are expressed during ovule primordia development. Of these, *CUC1* and *CUC2* play redundant roles in promoting ovule initiation in young gynecium with a fewer ovules [66]. *CUC2* and *CUC3* are also redundantly required for proper ovule development [30]. *SUP*, *SUB*, *KAN1/2,* and *TSO1* regulate late integument development and play a role in the genetic control of integument morphogenesis [40, 41, 43]. Of these, *KAN1* belongs to the KANADI family and is one of the most important genes in outer integument regulation [67]. In the current study, 29 DEGs related to ovule development were identified. Of these, the *AGL* genes regulate ovule morphogenesis; *ANT*, *CUC3,* and *WUS* regulate ovule primordia formation; and *ANT*, *BEL1*, *TSO1*, *SUB*, *SUP,* and *KAN1* regulate integument development. These genes presented different expression patterns in the ‘FT’ and *fsm* mutant plants; we speculated that the interaction and regulation of these genes may influence ovule development in the *fsm* mutants (Fig. 6).

Compared with the wild-type ‘FT’ plants, the *fsm1* mutant plants exhibited pistil abortion, and the floral organs were small. Plant organ size is determined by two developmental processes: cell proliferation and cell expansion [68]. In *A. thaliana*, the F-box protein SAP is a positive regulator of organ growth that regulates organ size by promoting cell proliferation [46, 51]. *ANT* not only regulates the formation of integument, but also regulates the size of plant organs [69]. *ANT* and *ARGOS* regulate organ size by influencing cell proliferation [61, 70, 71]. Loss-of-function *ARGOS* or *ANT* mutants exhibit smaller leaves and floral organs [53, 72]. *ANT* and *AIL6* are important regulators of floral development as they can regulate floral meristem and organ growth [73, 74]. *ANT*, *AIL5*, and *AIL7* play a redundant role in inflorescence meristem and flower development [75]. The TCP proteins are plant-specific transcription factors that can control leaf and flower size and shape [76]. In the current study, we identified 16 DEGs involved in the regulation of organ size, and they mainly included *ANT*, *ARGOS*, *TCP*, and *AIL*. The majority of these genes were downregulated in the *fsm1* mutant. The interaction of these organ size-related DEGs might result in the smaller floral organs observed in the *fsm* mutant plants (Fig. 6).

Hormones play an important role in the regulation of plant growth and development. Multiple mutant genes involved in hormone signaling pathways including the IAA, CK, ETH, GA, JA, and BR pathways have been previously determined in female sterile mutants, resulting in morphological abnormalities in the pistil [11, 34, 48, 77–80]. The transcriptome analysis of female sterile materials showed that DEGs are significantly enriched in the hormone signal transduction pathways in rice, pomegranate, and *Pinus tabuliformis*, indicating that changes in the hormone metabolic pathway have an important effect on ovule development [15–17]. Auxin plays an important role in ovule development and promotes carpel initiation and gynecium growth [81]. Furthermore, *ANT* has been proposed to act downstream of auxin in flower growth and patterning [73]. The auxin-response genes mainly include *ARF*, *GH3, SAUR,* and *AUX/IAA*. *ARF6* and *ARF8* are involved in ovule adaxial/abaxial polarity formation and play a vital role in ovule and anther development [17, 82]. The downregulation of *ARF6* and *ARF8* can result in female sterility [77]. CK positively regulates ovule formation and pistil development [83]. Furthermore, the *BEL1* transcription factors play an important role in cytokinin signaling pathways for correct ovule patterning [34]. Indeed, the CK levels have been positively correlated with ovule numbers. Reduced CK content results in a corresponding significant reduction in both ovule number and pistil size, thus resulting in female sterility [84–86]. ETH contributes to pistil development [78, 87] and ethylene-responsive factor (*ERF*) ethylene-response signal genes belong to the *AP2* gene family, which positively regulate ETH. The ERF protein can also inhibit *AG* gene expression, thus affecting ovule development [21, 88, 89]. BR plays a role in the development of the ovule outer integument and gynecial medial domain [79]. GA plays a major role in the control of ovule integument development and ovule initiation [90] and negatively modulates the number of ovules in plants [48]. JA plays an important role in determining the fate of pistils as high JA levels promote pistil abortion [11]. The functional enrichment analysis in the present study showed that the GO terms related to hormones were enriched and that the plant hormone signal transduction pathways were also significantly enriched. The DEGs identified in our study were involved in IAA, CK, ETH, GA, JA, and BR signaling. Of these pathways, the IAA signaling pathway had the highest number of DEGs. Further study of these genes involved in hormone signal transduction may elucidate the pistil abortion mechanism; different hormones may have synergistic or antagonistic effects in regulating pistil development in Chinese cabbage.

## Conclusions

The ovules play a major role in sexual reproduction, and they are the female reproductive organs in Chinese cabbage. Our study clarified the function of *BraA04g009730.3C* and revealed that it was responsible for ovule development and organ size. Comparative transcriptome analyses were performed to explore the regulatory effect of *SAP* in ovule development, and several DEGs related to ovule development were identified. Our study provides valuable information for future studies on pistil development and lays a solid foundation to elucidate the molecular mechanism of pistil development in Chinese cabbage.

## Methods

### Plant materials and mutagenic treatment

The wild-type ‘FT’ was a DH line derived from Chinese cabbage variety ‘Fukuda 50’, which was screened by Shenyang greenstar Chinese cabbage research institute (Shenyang, China) [91]. Germinated ‘FT’ seeds were immersed in 0.8% EMS solution for 12 h, and then thoroughly washed in running water for 12 h. After vernalization treatment at 2 °C for 15 d, the seeds were sown in a greenhouse in Shenyang Agricultural University. All live plants (M_0_ generation) were self pollinated. The mutant materials were screened and identified in the M_1_ generation to obtain the parallel female sterile mutant *fsm2*.

### Observation of morphological characteristics

In the full-bloom stage, floral organ characteristics were observed and compared between the *fsm1* and *fsm2* mutants and wild-type ‘FT’ plants. According to our previous method [44], three each of ‘FT’ and mutant *fsm2* plants were selected and artificial self-pollination of the *fsm2* mutant and a reciprocal cross between ‘FT’ and the *fsm2* mutant was performed. Seed-setting rates of each plant type were recorded and analyzed.

### Genetic analysis

Mutant *fsm2* and wild-type ‘FT’ plants were employed as parents to obtain the F_1_, F_2_, and BC_1_ populations. The phenotype of each plant in each generation was recorded to investigate the genetic characteristics of mutant *fsm2* plants.

### Allelism test between the *fsm1* and *fsm2* mutants

Both F_1_ (‘FT’ × *fsm1*) and F_1_ (‘FT’ × *fsm2*) populations exhibited the normal phenotype. The F_1_ (‘FT’ × *fsm1*) and F_1_ (‘FT’ × *fsm2*) populations were used as parent plants. Reciprocal crosses were made to obtain phenotypic segregation ratios of the populations. The population segregation ratios were analyzed using the Chi-square (χ^2^) test at the 0.05 level.

### Gene cloning and sequencing

The coding sequence of *BraA04g009730.3C* was amplified in mutant *fsm2* plants using the primer sequences shown in Additional file 8: Table S7. Gene cloning was performed according to the method of Huang et al. [92] and samples were sequenced by GENEWIZ (Suzhou, China) using the Sanger method. Sequences were aligned and analyzed using DNAMAN software. In addition, the online software SWISS-MODEL (https://swissmodel.expasy.org/) was used to analyze the three-dimensional protein structures of wild-type ‘FT’ and mutant *fsm2*.

### RNA extraction, cDNA library construction, and Illumina sequencing

In the full-bloom stage, five wild-type ‘FT’ and five *fsm1* mutants were selected. Pistils within the mature flower buds of ‘FT’ and mutant *fsm1* plants were randomly selected and mixed; the mixed samples were used as one biological replicate. Three independent biological replicates of both ‘FT’ and *fsm1* mutant were used.

The total RNA of the six samples was extracted using the TRIzol kit (Invitrogen, USA) following the manufacturer’s instructions. The quality and purity of the total RNA were checked using a NanoDropND-1000 spectrophotometer (NanoDrop, USA), and integrity was detected using the Agilent 2100 Bioanalyzer (Agilent, USA).

The six samples were designated as FT1, FT2, and FT3 (three biological replicates of ‘FT’) and M1, M2, and M3 (three biological replicates of mutant *fsm1*). Equal amounts of total RNA from the six samples were pooled for RNA-Seq library construction. The six cDNA libraries were sequenced using the Illumina novaseq-PE150 sequencing platform at Novogene (Beijing, China).

### RNA-Seq analysis, differential gene expression, and functional enrichment analysis

Clean reads were mapped to the *Brassica* reference genome (http://brassicadb.org/brad/datasets/pub/Genomes/Brassica_rapa/V3.0/) using HISAT2 software.

Fragments per kilo bases per million fragment (FPKM) values and DESeq software [93] were used to analyze differential gene expression. DEG screening criteria were defined as having a |log_2_(fold change)| > 1 and *P*-value < 0.05. Significantly enriched GO terms and KEGG pathways of DEGs were analyzed using the topGO and Kyoto Encyclopedia of Genes and Genomes (KEGG) databases, respectively.

### RT-qPCR analysis

Twenty-four genes related to ovule development were selected for the RT-qPCR analysis and the gene-specific primers were designed using Primer Premier 5.0 software. The primer sequences are listed in Additional file 9: Table S8. The cDNAs of ‘FT’ and mutant *fsm1* pistils (collected as described in RNA-Seq) were employed as templates for RT-qPCR using UltraSYBR Mixture reagent (CWBIO, China) and the QuantStudio 6 Flex Real-Time PCR System (ABI, USA). The reaction system and program were used according to the manufacturer’s instructions. The 2^-ΔΔCt^ method was used to calculate the relative gene expression levels [94]. *Actin* and *18S rRNA* were used as the internal controls [95]. All reactions were performed with three technical and biological replicates, and the data were analyzed using OriginPro8.0. Significant difference at the 0.05 level was determined using the *t*-test with SPSS 16.0 software.

## Supplementary Information

**Additional file 1: Table S1.** Summary of all expressed genes detected in the ‘FT’ and *fsm1* mutant libraries.

**Additional file 2: Table S2.** List of DEGs identified by comparing the *fsm1* mutants and ‘FT’ plants.

**Additional file 3: Table S3.** List of specifically expressed genes identified in *fsm1* mutants compared with those in the ‘FT’ plants.

**Additional file 4: Figure S1.** GO functional classification of DEGs between the *fsm1* mutants and ‘FT’ plants.

**Additional file 5: Table S4.** Significantly enriched GO terms identified in the *fsm1* mutants compared with those in the ‘FT’ plants.

**Additional file 6: Table S5.** Twenty most significantly enriched KEGG metabolic pathways.

**Additional file 7: Table S6.** DEGs involved in the plant hormone signal transduction pathways.

**Additional file 8: Table S7.** Primers for *BraA04g009730.3C* coding sequences.

**Additional file 9: Table S8.** Primer sequences used for the RT-qPCR analysis.

## Data Availability

The data charts supporting the results and conclusions are included in the article and additional files. Transcriptome sequencing data have been deposited in the NCBI Gene Expression Omnibus (GEO) Database under accession number GSE147438 (https://www.ncbi.nlm.nih.gov/geo).

## Abbreviations

EMS: Ethyl methanesulfonate
DH: Double haploid
IAA: Auxin
CK: Cytokinin
GA: Gibberellin
ABA: Abscisic acid
ETH: Ethylene
BR: Brassinosteroid
JA: Jasmonic acid
SA: Salicylic acid
SNP: Single nucleotide polymorphism
