# Supplementary figures and images for: The SAP function in pistil development was proved by two allelic mutations in Chinese cabbage (Brassica rapa L. ssp. pekinensis)
Source: BMC Plant Biol. 2020 Nov 30;20:538. doi: 10.1186/s12870-020-02741-5 (PMC7708145; doi:10.1186/s12870-020-02741-5)

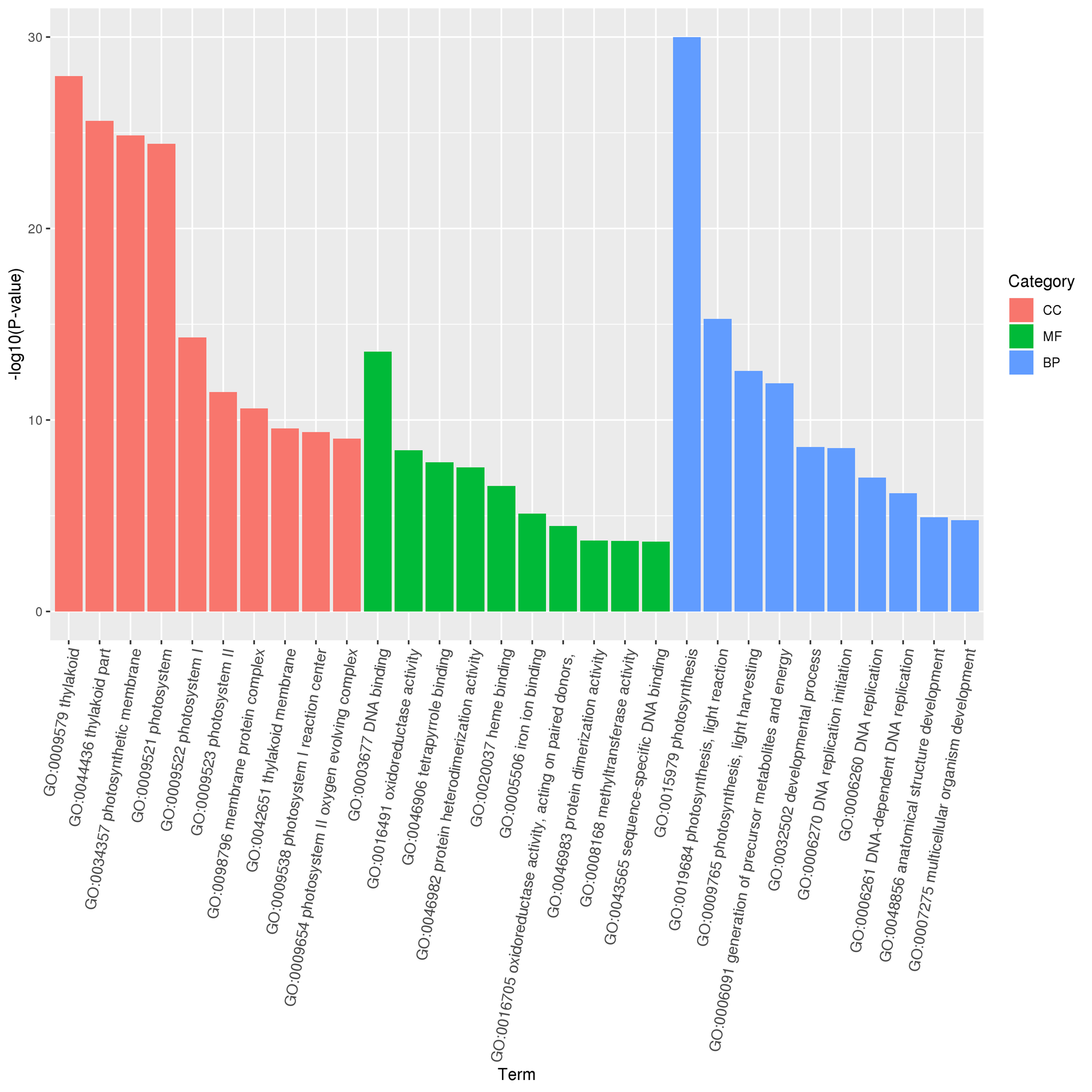

Supplement: Supplementary file 4 — Additional file 4: Figure S1. GO functional classification of DEGs between the fsm1 mutants and ‘FT’ plants. [file 12870_2020_2741_MOESM4_ESM.tif]
